# Diagnostic performance and survival outcome following sentinel lymph node biopsy in breast cancer patients from a tertiary cancer centre in India

**DOI:** 10.3332/ecancer.2022.1398

**Published:** 2022-05-26

**Authors:** Sanjit Kumar Agrawal, Himanshu Sharma, Noopur Priya, Anoop P Saji, Hamyung Denchu Phom, Abhishek Sharma, Indu Arun, Jayanta Das, Aditi Chandra, Rosina Ahmed

**Affiliations:** 1 Department of Breast Oncosurgery, Tata Medical Center, Kolkata 700156, India; 2 Department of Histopathology, Tata Medical Center, Kolkata 700156, India; 3 Department of Nuclear Medicine, Tata Medical Center, Kolkata 700156, India; 4 Department of Radiology, Tata Medical Center, Kolkata 700156, India

**Keywords:** breast cancer, sentinel lymph node biopsy, India

## Abstract

**Background:**

Sentinel lymph node biopsy (SLNB) has replaced axillary lymph node dissection (ALND) for axillary staging in early node-negative breast cancer (BC) patients in developed countries. However, in resource-constrained developing countries, adoption of SLNB is slow due to logistic issues and lack of outcome data from non-screened BC cohort. Therefore, we aim to report diagnostic performance, surgical morbidity and survival outcome of SLNB in BC patients from a tertiary care cancer centre in India.

**Methodology:**

1,521 consecutive early node-negative T1-3N0 BC patients having SLNB from 2011 to 2020 were included in the study. Data were retrieved from the institutional Redcap database and electronic medical records. Analysis was done using Stata14.

**Results:**

SLNB was done by dual dye (methylene blue (MB) + radioisotope (RI)/indo cyanine green (ICG)) in 57.7%, MB only in 39.3%, and RI alone in 3% of patients. The identification rate (IR) and SLNB positivity rate were 96% and 27.7%, respectively. IR was highest (98%) with MB + ICG and lowest (94%) with MB alone SLNB. UltraSonoGraphy guided fine needle aspiration cytology of radiological suspicious nodes has significantly reduced the SLNB positivity rate from 34.6% to 26.4% (*p* < 0.01). One patient had skin necrosis, and 16 had persistent blue staining of the skin in the MB injection site. All were managed conservatively. The lymphedema rate was significantly higher (5.2%) in the ALND versus 0.5% in the SLNB alone patients (*p* < 0.05). In a median follow up of 27 months, the axillary recurrence rate was 0.04% (4/1,023), and false-negative rate was 0.9% in SLNB negative patients. There were 35 recurrences and 25 deaths in SLNB negative patients, with 10 years predicted disease-free survival of 81% (95% CI 66% to 89%) and overall survival of 79% (95% CI 59% to 90%).

**Conclusions:**

SLNB should be offered as an axillary staging procedure to all eligible BC patients from developing countries to avoid the morbidity associated with ALND. Fluorescent dye can be used as an alternative for RI in a resource-constrained setup.

## Background

Breast cancer (BC) surgical management has changed from traditional radical surgeries to conservative surgeries with better quality of life and equivalent or non-inferior survival outcomes in the last 50 years [1]. In the majority of the patients, the surgery for the breast has changed from radical mastectomy to breast conservation [2], and for axillary staging, from axillary lymph node dissection (ALND) to sentinel lymph node biopsy (SLNB) [3, 4]. Currently, SLNB should be used for axillary staging in the early node-negative BC and selected group of node-positive patients post neoadjuvant chemotherapy (NACT) [5]. SLNB alone significantly reduces the morbidity of the ALND like seroma, shoulder stiffness, paraesthesia of the medial part of the arm and lymphedema [6, 7].

SLNB has replaced ALND for axillary staging in most BC patients in developed countries. However, in developing countries, many surgeons still do not offer SLNB because of lack of training, availability of radioisotope (RI) or frozen section and financial constraints [8]. For example, a survey conducted in India during a national BC congress showed that only 68.5% of the surgeons offer SLNB as an axillary staging procedure for node-negative early BC [9] mainly due to the logistics of RI availability and lack of training. To overcome the availability and logistic issues of radioactive dye SLNB, until recently, most surgeons in developing countries were doing SLNB using blue dye (methylene or patent blue) [8, 9]. However, there were concerns about using blue dye alone due to a low identification rate (IR) and high false-negative rate (FNR) [10]. Fluorescent indo cyanine green (ICG) dye has become an alternative to RI for SLNB in BC patients in the last 5 years. ICG dye SLNB is easy to use, of low cost and possible in a resource-constrained setting without nuclear medicine setup. With ICG, dual dye SLNB can be offered in all eligible patients without radioactive dye, which has given momentum to adopting this procedure in developing countries [11].

We started our SLNB program for early BC in 2011 and have done around 1,500 SLNB procedures in the last 10 years. In earlier years, we faced problems with RI cost and nonavailability and have done methylene blue (MB) dye only SLNB frequently. With the introduction of ICG dye for SLNB in 2018 in our centre, dual dye (MB + ICG) SLNB could be done in the majority of the patients. There is a plethora of evidence for diagnostic and survival outcome of SLNB procedure from the screened BC patients in the western countries [3, 4, 12]; however, similar evidence in a large non screened BC patients cohort from developing countries is lacking.

The study aims to report the diagnostic performance and survival outcome of SLNB in a large non-screen detected BC patients’ cohort from a lower middle-income country (LMIC).

## Methodology

The institutional review board has approved the study with a waiver number EC/WV/TMC/12/21. It was a single centre retrospective study and included all consecutive patients diagnosed with BC and have undergone SLNB from 2011 to 2020 at the Tata Medical Center, Kolkata, India. SLNB was done for node-negative early breast cancer (EBC) patients, mainly as an upfront surgery and additionally, from July 2019 in the post NACT setting. As per institutional protocol, SLNB was offered to all cT1-3N0 BC patients. SLNB was not offered to patients with locally advanced and recurrent BC. From 2018, fine needle aspiration cytology (FNAC) of suspicious axillary nodes reported by UltraSonoGraphy (USG) axilla in clinically node-negative patients was performed to confirm node-negative status before offering SLNB. We performed ALND in patients with FNAC proven node-positive BC.

Data were retrieved from the institutional Recap database and electronic medical records. Our primary objective was to evaluate the diagnostic performance of the SLNB using IR, FNR and SLNB positivity rates. Other endpoints were to assess quality indicators of SLNB, comparative performance of different methods of SLNB used, adverse reactions of MB dye injection, lymphedema rate and survival outcome (isolated axillary recurrences, disease-free survival (DFS) and overall survival (OS)).

### Sentinel lymph node biopsy

SLNB was performed according to the institutional protocols, with the different dye combinations as per radioactive dye availability. Between 2011 and 2017, MB dye only was used when Tc-99 was not available. From July 2018, ICG based SLNB was introduced to overcome the problems of the non-availability of RI. Tc-99 (microfiltered (0.22) micron 99m Tc-S) injection and gamma probe (Europrobe3, EURORAD S.A2, Ettore Bugatti, 67201 ECKBOLSHEIM – FRANCE) were used to localise the hot nodes. 2 ml of 1% MB dye was used for blue dye SLNB. ICG (aurogreen) 1 ml (2.5 mg) injection and an infra-red camera (Irillic. nm PVT limited) were utilized to identify the fluorescent nodes. The node with maximum radioactivity and any other hot nodes showing counts more than ten times the background count, visibly blue-stained, fluorescent and clinically suspicious nodes were considered SLNs. The SLNs were sent for frozen section examination.

### Histopathological examination

Frozen section analysis was performed as per the ASCO CAP guidelines. Lymph nodes were thinly sliced at 2 mm intervals along the longitudinal axis, and opposing surfaces were submitted to frozen section examination if the lymph node was bisected. If >3 slices were made, the non-opposing surfaces of middle slices were submitted for frozen section evaluation so that the tissue sections examined are not more than 2 mm away to prevent missing a macrometastatic deposit. Grossly negative lymph nodes were submitted entirely, while representative sections of grossly positive nodes were submitted for microscopy. If the initial (H and E) stained section was negative for metastasis, two level sections were examined. Frozen section remnants were entirely submitted for permanent section evaluation. Immunohistochemistry with an anti-cytokeratin antibody cocktail (cytokeratin AE1–AE3; Dako Corporation, Denmark) was performed in tissue sections when metastatic carcinoma was suspected morphologically.

Macro metastasis was defined as a single focus of metastasis measuring >2 mm in a given lymph node. Micro metastasis measured more than 0.2 mm, but not more than 2 mm. The presence of isolated tumour cell clusters (ITCs) was defined as single cells or small clusters of cells not larger than 0.2 mm and no more than 200 cells in a single crosssection.

### ALND and adjuvant treatment

ALND was done in all patients with SLNB positive with macrometastasis. From March 2017 onwards, patients with micrometastatic SLNs did not have ALND. SLNs positive with isolated tumour cell (ITC) only were considered node-negative, and ALND was not done. Adjuvant chemotherapy, radiotherapy, endocrine and targeted therapy were advised as per the multidisciplinary team plan and institutional protocol.

### Outcome assessment

Blue staining of the skin and necrosis at the site of MB dye injection were recorded at the time of the discharge and post-op outpatient department visit assessments. Lymphedema of the arm was evaluated by clinical examination at baseline, and each follow up visit. Isolated axillary recurrence was defined as biopsy/FNAC proven metastatic recurrent axillary node with no metastasis elsewhere on whole-body PET CT examination. The DFS was defined as the duration between the date of diagnosis and the date of recurrence/death/last follow-up date. Overall survival was defined as the duration between the date of diagnosis and date of death due to any cause/last follow up. The following formulae measured SLNB performance and quality metrics:

- IR: Number of patients with SLN identified/total sample size
- FNR: False-negative (number of patients with axillary recurrence)/false-negative + true positive
- SLNB positivity rate: Number of patients with metastatic SLN/number of patients with SLN identified

### Statistical analysis

The data normalcy was checked by the Shapiro–Wilks test. The comparison between categorical variables was made by chi-square/Fisher exact test as applicable. For continuous variables comparisons, Student *t* and one way analysis of variance tests were used. Survival analysis was done by Kaplan–Meier graphs and estimation. All *p* values were two-tailed, and values <0.05 were considered to be significant. Analysis was done using Stata 14 version software.

## Results

The mean age and body mass index were 55 (SD = 12) years and 27.3 (SD = 4.8) kg/m^2^. The median tumour size was 2.8 (SD = 1.3) cm. The majority of the patients had invasive ductal cancer (78.8%), T2 (75.2%) and Grade 3 (59.2%). In T3 patients, only 6/58 have received neoadjuvant therapy. The majority of T3 patients were elderly (mean age 63 (SD 11)) and have chosen upfront mastectomy (82%) as an option. Breast conservation surgery was done in 60% of patients. SLNB was done along with breast tumour surgery in 95% of patients. The SLNB IR was 96%, and the SLN positivity rate was 28.7%. Demographic and clinicopathological characteristics are summarised in Table 1.

SLNB was done by dual dye (MB + RI/ICG) in 57.4% of patients. The diagnostic performance and quality indicators of four different methods of SLNB (MB only, RI only, MB + RI and MB + ICG) are summarised in Table 2. The MB + ICG method had the highest IR of 98%; however, this method was also associated with an increased number of SLN retrieval (3.4 (SD 1.8)). The SLNB positivity rate was lowest (25%) with MB + ICG method. More than 1 SLNs were removed in 82% of the patients, and SLN was the only positive node in 57% of patients.

From July 2018, there were two significant changes in institutional protocol, with the introduction of ICG method of SLNB and pre-operative USG guided FNAC of all suspicious axillary nodes. Therefore, we have analysed the data in two time frames: 2011–7 versus 2018–20 (Table 3). With the introduction of ICG, the dual dye method was used in 74.5% in 2018–20 compared to 36.5% in 2011–7. The SLNB positivity rate was 26.4% in 2018–20 compared to 34.6% in 2011–7.

Adverse reactions to MB dye, lymphedema rate and survival outcomes of SLNB negative patients are summarised in Table 4. Out of 1,471 patients in whom MB was injected, 1 had skin necrosis, and 16 patients had bluish discolouration of the skin 1 week after surgery. The lymphedema rate was 5.2% in the ALND group and 0.5% in the SLNB only group. The lymphedema rate was significantly associated with radiotherapy (*χ*^2^ = 4.8, *p* = 0.04). The type of surgery (breast conservation surgery (BCS)/mastectomy) was not associated with the lymphedema (*χ*^2^ = 0.6, *p* = 0.4).

The compliance to adjuvant treatment like chemotherapy, radiotherapy and endocrine therapy was >95% in our cohort. Unfortunately, due to financial constraints, only 108/253 (42.3%) of HER 2 positive BC patients received trastuzumab. In a median follow up of 27 months, 4/1,023 SLNB negative patients had isolated axillary recurrence (AR rate of 0.4%). Considering axillary recurrence as a false negative SLNB procedure, the FNR was 0.9%. There were in total 35 recurrences and 25 deaths in SLNB negative patients, with 10 years predicted DFS of 81% (95% CI 66% to 89%) and OS of 79% (95% CI 59% to 90%). Among HER 2 positive patients, trastuzumab therapy was not significantly associated with DFS (log-rank *p* = 0.67) and OS (log rank *p* = 0.08).

## Discussion

To the best of authors’ knowledge, this is the largest study from an LMIC reporting the diagnostic performance and survival outcome of SLNB in node-negative early BC patients. The IR, FNR, DFS and OS are comparable to published results from the seminal randomised controlled trials of SLNB in BC. Moreover, the diagnostic performance of different methods of SLNB, even MB alone, were in an acceptable range. For example, the IR was >90% with any of the four methods used for SLNB. The side effects of MB dye injections, like skin necrosis and hypersensitivity reaction, were minimal in our series. The lymphedema rate was significantly lower in BC patients with SLNB only compared with those having ALND.

The SLNB technique is mainly assessed by the IR, FNR, isolated axillary recurrence rate (ARR), DFS and OS. In our study, the IR, FNR, ARR, 10 years predicted DFS and OS were 96%, 0.9%, 0.4%, 81% (95% CI 66% to 89%) and 79% (95% CI 59% to 90%) respectively. These figures are in a similar range to the results of major randomised controlled trials (RCTs) of SLNB versus SLNB + ALND in EBC patients, which report IR > 95%, FNR < 10%, ARR < 1%, DFS of 81%–90% and OS of 89%–95% in the long term follow up [3, 4, 13]. Although median follow up time was 27 months in our study, ARR of 0.4% is reassuring as two major studies, NSABP04 and Z0011, have shown that an axillary recurrence is an early event with more than 80% of isolated axillary recurrences events happened in the first 2 years of follow up in patients whom ALND was omitted [14, 15]. The 10 years predicted DFS and OS in SLNB negative patients were marginally lower in our cohort, which may be due to a higher T stage in our non-screen detected BC cohort, which comprises mainly T2 tumours (75%). The 30% SLNB positivity rate was higher in our cohort than 20% in the published series as, until 2018, patients with radiologically suspicious nodes often underwent SLNB without prior FNAC. From 2018 onwards, we have seen a significant decrease in SLNB positivity rate as all clinically negative and USG suspicious axillary LN had FNAC, and we did not offer SLNB to patients with FNAC proven metastatic axillary nodes (Table 3).

We started our SLNB program with radioactive dye in 2011 and have faced the challenge of radioactive dye use in developing countries, as reported before [11]. Initially, we offered MB dye only SLNB when Tc-99 service was not feasible. From 2018, we started using ICG dye for SLNB, and dual dye SLNB could be done in the majority of the patients. We have compared the diagnostic performance of different methods of SLNB used, and IR was highest (98%) with ICG + MB dye and lowest (94%) with MB alone. In the last decade, SLNB by ICG alone in combination with blue dye has emerged as an alternative to RI, with the best diagnostic performance in terms of IR of 98%–100% [11, 16]. The meta-analysis of studies having SLNB by MB dye only in BC patients has shown an acceptable IR of 91%. However, cautioned generalising this technique due to a high FNR of 13% [10]. The mean number of SLN removed was highest in the MB + ICG group, due to rapid uptake in the lymphatics, and this is a reported drawback of fluorescent dye technique [17]. In our study, the SLNB positivity rate was lowest with ICG, which may be due to two changes of clinical practice in a similar time frame – introduction of ICG and USG guided FNAC for radiologically suspicious nodes to exclude node-positive patients from SLNB.

The most commonly used blue dyes for SLNB in BC are patent blue, isosulfan blue and MB. Patent and isosulfan blue are associated with adverse reactions, ranging from blue hives to wheals and flares to severe anaphylaxis in around 1% of patients [18]. In comparison, MB dye has been reported safer with regards to anaphylactic reaction [10]. In our large series, we did not notice a single hypersensitivity reaction to MB dye. Although MB dye is safer and not associated with life-threatening hypersensitivity reactions, some authors have reported skin staining and necrosis of the local injection area [19, 20]. In our series, 1 patient had skin necrosis which was managed by debridement and secondary skin suturing, and 16 patients had bluish stating of the injection site beyond 1-week post-surgery. The post-surgical morbidity like shoulder stiffness, lymphedema rate, tingling and paraesthesia are significantly lower in SLNB compared to ALND in the large multicentric RCT studies [6, 21]. We found a significant lower lymphedema rate of 0.5% following SLNB alone compared to 5.2% in ALND patients. Compared to published studies, the lower lymphedema rate in the both SLNB and ALND arm may be due to the shorter follow up duration in our series.

We have summarised major published studies from India, focusing on SLNB use in early BC patients in Table 5 [11, 22–32]. In summary, the SLNB procedure was done by different methods using blue, radioactive and fluorescent dyes. The majority are validation studies with the IR ranging from 77% to 100%, FNR 2.6% to 16.6% and SLNB positivity rate of around 30%. However, there is little available data on outcome for ipsilateral axillary recurrence, and the sample size for most studies is small. Our study of 1,521 patients with 0.4% ARR in 27 months of follow-up may set a benchmark for widespread adoption of SLNB by patients and surgeons in developing countries.

This study has some potential limitations. Being a retrospective study, the results may be associated with selection biases. The follow-up duration of 27 (13, 48) months, may be inadequate to compare survival and post-surgical morbidity outcome between SLNB and ALND patients. The adjuvant treatment details were brief and limited in our database. In our cohort >50% of HER 2 positive patients have not received trastuzumab, which must be considered in surgery de-escalation decision making, as long-term outcomes may be affected by the incomplete treatment. The quality of life measures and patient-reported outcomes were not recorded, which may be done in future studies with the same cohort of patients.

## Conclusion

This study provides evidence of the use of SLNB in non-screened early node-negative BC patients. It highlights the different possible methods of SLNB, and any one of them can be used as per the local resources. Fluorescent ICG dye may be a good alternative to the RI, and in combination with MB to offer dual dye SLNB in BC patients. Minimal axillary surgery in the form of SLNB is technically feasible in low resource settings and should be offered to all eligible patients to improve quality of life with equivalent or non-inferior survival.

## Conflicts of interest

The authors declare that they have no conflicts of interest.

## Funding

This study did not receive any specific grant from funding agencies in the public, commercial, or not-for-profit sectors.

## Figures and Tables

**Table 1.** Demographic and clinical characteristics.

Parameters (*n* = 1,521)	*n* (%)
**Type of cancer**	
DCIS	77 (5.1%)
IDC	1,199 (78.8%)
ILC	69 (4.5%)
Others	176 (11.6%)
**T stage**	
Tis	77 (5.1%)
T1	241 (15.9%)
T2	1,145 (75.2%)
T3	58 (3.8%)
**Grade (*n* = 1,428, ex DCIS)**	
Grade 1	83 (5.8%)
Grade 2	499 (34.9%)
Grade 3	846 (59.2%)
**ER (*n* = 1,442, ex DCIS)**	
Positive	1,105 (76.6%)
Negative	337 (23.4%)
**PR (*n* = 1,442, ex DCIS)**	
Positive	996 (69%)
Negative	446 (31%)
**HER 2 (*n* = 1,436, ex DCIS)**	
Positive	246 (17.1%)
Negative	841 (58.6%)
Equivocal	349 (24.3%)
**First treatment**	
Surgery	1,444 (95%)
NACT/NAET	77 (5%)
**Surgery**	
BCS	910 (60%)
Mastectomy	611 (40%)
**SLNB methods**	
MB only	598 (39.3%)
RI only	46 (3%)
MB + RI	320 (21%)
MB + ICG	557 (36.7%)
**SLNB**	
Not identified	61 (4%)
Negative	1,023 (67.3%)
Positive	437 (28.7%)
**Type of SLN metastasis**	
ITC	19 (4.3%)
Micrometastasis	58 (13.3%)
Macrometastasis	360 (82.4%)
**Axillary staging**	
SLNB negative (no ALND)	1,023 (67.3%)
SLN positive (no ALND)	33 (2.2%)
SLNB not identified (ALND)	61 (4%)
SLN positive (ALND)	404 (26.5%)

DCIS: Ductal Carcinoma In Situ, IDC: Invasive ductal cancer, ILC: Invasive lobular cancer, ER: Oestrogen receptor, PR: Progesterone receptor, MB: Methylene blue, NACT: Neoadjuvant chemotherapy, NAET: Neo adjuvant endocrine therapy, BCS: Breast conservation surgery, RI: Radioisotope, ICG: Indo cyanine green

**Table 2.** SLNB by different methods.

	Study sample	MB only	RI only	MB + RI	MB + ICG	Statistical association
IR	1,460/1,521 (96%)	563/598 (94%)	43/46 (93.5%)	307/320 (96%)	547/557 (98%)	*χ*^2^ = 13.3, *p* = 0.004
Number of SLN removed, mean (SD)	3 (1.7)	2.7 (1.5)	2.3 (1.4)	2.8 (1.7)	3.4 (1.8)	*f* = 21.6, *p* = <0.001
SLNB positivity rate	437/1,460 (30%)	186/563 (33%)	16/43 (37%)	99/307 (32%)	136/547 (25%)	*χ*^2^ = 11.16, *p* = 0.01

MB: Methylene blue, RI: Radioisotope, ICG: Indo cyanine green, IR: Identification rate

**Table 3.** SLNB performance by years: 2011–7 versus 2018–20.

	2011–7	2018–20	Statistical association
**Method**			
MB only	374 (57%)	224 (25%)	*χ*^2^ = 636, *p* < 0.001
RI only	43 (6.5%)	3 (0.5%)	
MB + RI	232 (35.3%)	88 (11%)	
MB + ICG	8 (1.2%)	549 (63.5%)	
**IR**	624/657 (95%)	836/864 (96.7%)	*χ*^2^ = 3.1, *p* = 0.07
**Number of SLN removed, mean (SD)**	2.68 (1.5)	3.2 (1.8)	*t* = −6.5, *p* < 0.01
**SLNB positivity rate**	216/624 (34.6%)	221/836 (26.4%)	*χ*^2^ = 11.4, *p* < 0.01

MB: Methylene blue, RI: Radioisotope, ICG: Indo cyanine green, IR: Identification rate

**Table 4.** Surgical complications and survival outcome (median follow up = 27 (13, 48) months).

Parameters	*n* (%)	
**Blue skin staining 1 week after surgery**	16/1,475 (1%)	
**Skin necrosis**	1/1,475 (0.06%)	
**Lymphedema**		
SLNB only	6/1,057 (0.5%)	*χ*^2^ = 36, *p* < 0.001
ALND	24/458 (5.2%)	
**SLNB negative (*n* = 1,023)**		
Isolated axillary recurrence	4/1,023 (0.04%)	
FNR	4/441 (0.9%)	
DFS (10 year estimated)	81% (95% CI 66%–89%)	
OS (10 year estimated)	79% (57%–90%)	

**Table 5.** SLNB in early BC studies published from India (sample size > 50).

Study	Type of study	*N*	Dye used	IR (%)	FNR with validation ALND/AR (%)	SLNB positivity rate (%)	ARR
Parmar [22]	Validation	100	ISB	77	16.6	30	-
Deo [23]	Validation	76	ISB	90.8	15.7	17.3	-
Somsekhar [24]	Validation	100	ISB + Tc-99	100	3.7	27	-
Seenu [25]	Validation	119	PBV + MB + Tc-99	97.4 (MB + Tc-99) 96.6 (PBV + Tc-99)	2.56 (MB + Tc-99) 7.69 (PBV + Tc-99)	32.7 (MB + Tc-99) 20.87 (PBV + Tc-99	-
Rao [26]	Outcome	151	MB	100	-	19.8	0% at 10 months
Sanjit Agrawal [11]	Outcome	207	Tc-99 + MB versus ICG + MB	95 Tc-99 + MB 97 ICG + MB	-	31.3	-
Somashekhar [27]	Outcome	100	Tc-99, MB and ICG	100	-	32	-
Vikas Gupta [28]	Validation	60	MB/Tc-99 + MB	100	8.6 (MB) 4.3 (Tc-99 + MB)	11.6	-
Challa [29]	Validation	523	MB/Tc-99 + MB	91.3	8.4	29.4	-
Sreekar Devarakonda [30]	Outcome	172	MB	95.9	-	23.6	0% at 26.68 ± 15.9 months
Gaurav Agrawal [31]	Validation	78	MB + Tc-99 Antimony/MB + Tc-99 Sulphur	MB + Antimony 100 MB + Sulphur 97.4	MB + Antimony 6.3 MB + Sulphur 7.7	35	-
Parmar [32]	Validation	478	MB + Tc-99	93.5	12.7	34.1	-
Present study	Outcome	1,521	MB/Tc-99/MB + Tc-99/MB + ICG	96	0.9%	30	0.4% at 27 (13, 48) months

ISB: Isosulphan blue, PBV: Patent blue V, MB: Methylene blue, IR: Identification rate, FNR: False negative rate, ARR: Axillary recurrence rate
